# Tumor-infiltrating T lymphocyte clonality predicts prognosis in human ovarian cancer

**DOI:** 10.1186/2051-1426-1-S1-P64

**Published:** 2013-11-07

**Authors:** Takemasa Tsuji, Kevin H Eng, Sacha Gnjatic, Junko Matsuzaki, Rachel Brightwell, Anthony Miliotto, Ryan Emerson, Cindy Desmarais, Erika Lindsley, Julie Rubinstein, Jianmin Wang, Song Liu, Harlan Robins, Kunle Odunsi

**Affiliations:** 1 Center for Immunotherapy, Roswell Park Cancer Institute, Buffalo, NY, USA; 2 Biostatistics and Bioinformatics, Roswell Park Cancer Institute, Buffalo, NY, USA; 3 Tisch Cancer Institute, Icahn School of Medicine at Mount Sinai, New York, NY, USA; 4 Gynecologic Oncology, Roswell Park Cancer Institute, Buffalo, NY, USA; 5 Adaptive Biotechnologies, Seattle, WA, USA

## Background

The prognostic significance of the number of tumor-infiltrating T cells has been demonstrated for many tumor types. In contrast, the significance of the tumor-infiltrating T cell clonality, which reflects the preferential infiltration or expansion of T cell clones in the tumor microenvironment, has not been clear because of the technical hurdles required for evaluating each T cell clone in the tumor. In order to delineate the complexity of T cell responses and define correlates of a protective immunity, we applied a recently developed deep T cell receptor (TCR)-sequencing technology (immunoSEQ) to paired frozen tumor tissues and peripheral blood mononuclear cells from 99 ovarian cancer patients.

## Methods

Rearranged β TCR chain DNA sequences were sequenced using immunoSEQ technology. Frequency of each T cell clone was obtained from the copy number of the sequence. T cell clonality of the specimens was calculated from entropy of TCR sequences. Spontaneous immune responses against tumor-associated antigens (NY-ESO-1, MAGE-A1, MAGE-A3 and p53) were evaluated by measuring serum antibodies by ELISA.

## Results

Approximately 3(±3)×10^6^and 4(±2)×10^6^ full-length TCR beta chain sequences were obtained corresponding to the detection limit for T cell frequency at 3×10^-7^and 2×10^-7^ for tumor and blood samples, respectively. In patients who had spontaneous antibodies against a panel of tumor-associated antigens, more clonal T cell infiltration was associated with longer progression-free survival. In sharp contrast, clonal infiltration was a worse prognostic factor in patients without detectable humoral immune responses against surrogate tumor antigens. From sequence based analyses, we found a set of shared TCR sequences among patients.

## Conclusion

Deep TCR sequencing using immunoSEQ technology is a powerful tool to characterize tumor-infiltrating T cell clonality using frozen tumor tissues. Our analyses indicate that evaluation for spontaneous anti-tumor immune responses is required to correctly understand the prognostic significance of tumor-infiltrating T cells.

